# Coincidence of Acute Brucella Hepatitis and Dengue Fever or Serologic Cross-reactivity?

**DOI:** 10.4103/1319-3767.70621

**Published:** 2010-10

**Authors:** Khalid I. Bzeizi, Ali Benmousa, Faisal M. Sanai

**Affiliations:** Division of Gastroenterology and Hepatology, Department of Medicine, Riyadh Military Hospital, Riyadh, Saudi Arabia

**Keywords:** Brucellosis, cross-reactivity, dengue fever, hepatitis, serum transaminase

## Abstract

Hepatic involvement in brucellosis is not uncommon since 10-20% of patient infected with brucella species can have abnormal liver function tests. The usual presentation of brucella hepatitis is in the form of chronic granulomatous hepatitis with mild to moderate elevation of liver enzymes, while acute hepatitis is rare. We report a young patient who presented with acute brucella-induced hepatitis and co-infection with dengue hemorrhagic virus resulting in severe elevation of liver enzymes and absence of granuloma on histology. His mother also simultaneously tested positive for both infections. The patient responded well to anti-brucella therapy with normalization of his liver profile. We discuss, herein, the hepatic involvement of these two infections and discuss the possible serological cross-reactivity between brucella and dengue fever virus.

Brucellosis is a febrile zoonotic infectious disease with occasional hepatic involvement in 10–20% of cases.[1,2] Hepatic involvement usually manifests as a non-specific liver enzyme elevation, while granuloma within the liver may commonly occur.[1,3] Similarly, dengue fever is an acute febrile illness caused by a flavivirus and may in turn cause liver enzyme abnormalities. Co-infection of these two pathogens, while feasible, has not been previously described. We describe in this report the simultaneous serological documentation of these two pathogens suggesting an acute infection in two members of the family concomitantly.

## CASE REPORT

A 23-year-old Saudi male, previously fit and well, presented with 3 days of fever, chills, epigastric pain, jaundice, and anorexia. Three weeks prior to the onset of illness, he had returned to Saudi Arabia from Pakistan. He denied any history of blood transfusion, use of herbal medications, raw milk ingestion, and contact with animals or with any jaundiced patient. He also denied alcohol/drug abuse or extra-marital sexual relationship. His mother was diagnosed earlier with brucellosis and had just commenced treatment when he returned from Pakistan.

On examination, he looked unwell, was jaundiced and febrile (39.7°C). He had bilateral sub-conjunctival hemorrhages but no evidence of bleeding tendency elsewhere. The rest of his examination was unremarkable.

The initial laboratory investigations showed a total bilirubin of 225 μmol/L (normal 2–22), albumin 42 g/L (normal 36–51), alkaline phosphatase (ALP) 146 U/L (normal 35–129), alanine transaminase (ALT) 1587 U/L (normal 2–40), aspartate transaminase (AST) 1438 U/L (normal 2–37), and gamma glutamyl transpeptidase (GGT) 40 U/L (normal 11–50). Full blood count and coagulation profile were normal. His viral screen was positive for anti-hepatitis A virus (HAV) IgG and anti-hepatitis E virus (HEV) IgG, while HAV and HEV IgM, HBsAg, anti-HBV IgM and hepatitis C virus serology as well as HCV RNA were all negative. Other viral screen including HIV, cytomegalovirus, and Epstein-Barr-Virus were negative as well. Blood film for malaria was negative several times and auto-antibodies (antinuclear and anti-smooth muscle antibodies) were not detected.

An ultrasound of the abdomen with Doppler was completely normal. Blood culture was positive for *Brucella melitensis* and brucella serology was strongly positive and therefore he was started on doxycyclin and gentamycin. Screening test for viral hemorrhagic fever was done upon his arrival because of the presence of fever, subconjunctival hemorrhage, and living in two endemic areas. The test was strongly positive for dengue IgM with a titer of 1:2560. Liver histology showed intact architecture with heavy infiltration of the portal tracts by a mixture of mainly neutrophils and eosinophils. There was prominent canalicular cholestasis and Kupffer cell hyperplasia but no granulomas were seen.

The patient showed significant improvement both clinically and biochemically after starting anti-brucella treatment. His liver profile returned to normal values within 6 weeks from the commencement of treatment.

We opted to test the patient’s mother for dengue fever in view of the above findings. Serological tests were done on a previously stored sample taken at the start of her illness which revealed strong dengue fever seropositivity (IgM) with a titer of 1:1280.

## DISCUSSION

Brucellosis is a febrile zoonotic infectious disease that has a tendency toward chronicity and is capable of multisystem involvement.[4] It is caused by facultative intracellular bacteria that can multiply within phagocytic cells in different tissues. In many developed countries, brucellosis is considered an endemic disease and although rarely fatal, it is associated with significant morbidity to the infected host.[5] In Saudi Arabia, brucella is reported as endemic in many regions including the central, north, and south regions.[6,7]

Hepatic involvement in brucellosis is seen in 10 - 20% of patients.[1,2] Hepatomegaly is the most commonly reported hepatobiliary sign which occurs in up to one to two-thirds of patients,[8] although not always associated with abnormality in liver function. Hepatic inflammation was described in 10% of a University of Minnesota brucella series of 244 patients.[1] The level of ALT elevation in brucella hepatitis is described as modest and in majority of the patients is less than two to three times the upper limit of normal.[9]

Our patient has a definite active infection with *Brucella melitensis* as proven by positive blood culture and very high brucella titer and he had evidence of acute hepatitis clinically, biochemically, and histologically. Acute hepatitis, although a recognized feature of brucellosis, is however, very rare and only a few cases so far have been reported in the literature.[9,10]

Hepatic granuloma is relatively common in patients with chronic brucellosis,[1] while not being universal.[11] While our patient’s hepatic histology did not reveal granuloma, his level of transaminase elevation was also much higher than what has been described in brucella hepatitis. Pakistan is endemic for dengue hemorrhagic fever, and an epidemic was reported in the Western region of Saudi Arabia during the period of the patient’s illness. We performed dengue fever serology in our patient due to his atypical presentation for brucellosis and the presence of subconjunctival hemorrhages.

Dengue is a febrile illness that is caused by flavivirus. Hepatic involvement in patients with dengue fever varies from mild elevation in one or more liver enzymes to acute hepatic failure which is rare.[12,13] In a series of 1585 patients reported by the Dengue Reference Center (CRD) in Brazil, 44% had altered level of at least one of the enzymes (AST and/or ALT); 17% had at least one of the enzymes increased to three folds and 3.8% had acute hepatitis (ALT/AST level increased more than 10 folds).[14] The disease, however, is self-limiting and complete recovery is the rule.[14]

The presence of high titer of IgM antibodies against dengue in this patient makes it plausible to attribute his illness to this pathogen. However, we feel that the likely cause for hepatitis here is the brucellosis. His mother presented with the typical features of brucellosis and her diagnosis was confirmed both by culture and serology. She too had a high titer of IgM antibodies against dengue. She responded well to antibiotics with complete resolution of her symptoms in much the same way as her son.

A recent study in 800 blood donors from Mexico reportedly found that 59% of donors were reactive for anti-dengue virus IgG, and 0.71% was reactive for anti-brucella serology, without mentioning whether any patient showed common sero-positivity.[15] Moreover, none had clinical features of acute brucellosis or dengue fever, while only 2% of the overall samples were reactive for anti-dengue virus IgM antibodies. It is very unlikely that our patient and his mother both had brucellosis and dengue fever simultaneously. The positive culture for brucellosis in both patients would make brucella the more likely etiological agent rather than dengue fever. An explanation for this phenomenon is the possible cross-reactivity between brucella and dengue fever virus. While cross reactivity between brucella and other pathogens like Entameba histolotica had been suggested,[16] and similarly sera from patients with positive brucella serology were found to exhibit positivity against *E. coli* O157 and *Y. enterocolitica* O9,[17] there have been no reports of such cross-reactivity with viruses, and in particular with flaviviruses. On the other hand, antibodies from the sera of dengue fever patients can cross react with other flavivirus species including Japanese encephalitis and yellow fever viruses,[18] although no prior reports exist of cross reactivity with brucella.
